# Adjusting the Canadian Healthcare System to Meet Newcomer Needs

**DOI:** 10.3390/ijerph19073752

**Published:** 2022-03-22

**Authors:** Ginny Lane, Hassan Vatanparast

**Affiliations:** 1 Margaret Ritchie School of Family and Consumer Sciences, University of Idaho, Moscow, ID 83844, USA; 2 College of Pharmacy and Nutrition, School of Public Health, University of Saskatchewan, Saskatoon, SK S7N 4Z2, Canada; vatan.h@usask.ca

**Keywords:** immigrant health, refugee health, healthcare access

## Abstract

Newcomers’ ability to access healthcare can be impacted by cultural, religious, linguistic, and health status differences. A variety of options are available to support the development of healthcare systems to equitably accommodate newcomers, including the use of basic English and other languages in public health information, engagement with immigrant communities to advise on program development, offering culturally competent health services, interpretation services, and through creating space to collaborate with traditional practitioners. This study employed in-depth interviews with newcomer families from the Healthy Immigrant Children Study that had been living in Regina or Saskatoon, Saskatchewan, Canada, for less than 5 years, as well as with healthcare providers and immigrant service providers to understand how to improve healthcare services. Analysis of participant quotes related to accessible healthcare services revealed five main themes: (1) responsive, accessible services, (2) increasing cultural competence, (3) targeted newcomer health services, (4) increasing awareness of health services, and (5) newcomer engagement in planning and partnerships. An accessible healthcare system should include primary healthcare sites developed in partnership with newcomer service organizations that offer comprehensive care in a conveniently accessible and culturally responsive manner, with embedded interpretation services. The Saskatchewan healthcare system needs to reflect on its capacity to meet newcomer healthcare needs and strategically respond to the healthcare needs of an increasingly diverse population.

## 1. Introduction

The first universal, comprehensive, government-controlled medical insurance plan in North America, Medicare, was actualized in Saskatchewan on 1 July 1962 [1]. Medicare primarily covered hospital and physician services. According to the 1961 census, the Saskatchewan population (925,000) was largely of European descent, except for about 31,000 First Nations and 5000 Asians [2]. Given the majority population, Medicare was shaped to respond to Europeans’ healthcare needs and expectations. Over the years, insured healthcare services expanded to include new services, such as physiotherapy [3], and new technologies, such as Magnetic Resonance Imaging [4], as the understanding of healthcare needs broadened and technological advancements occurred.

In recent years, Saskatchewan has experienced dramatic demographic shifts primarily due to changing immigration patterns. In 2016, about 11% of the Saskatchewan population were visible ethnic minorities [5]. This figure will continue to grow as recent immigrants commonly originate from Asia and the Middle East [6]. Between 2011 and 2016, Saskatchewan experienced the largest percentage increase in the immigrant population across Canada (74%, 47,935 immigrants over 5 years in a province with population of just over 1 million) [7], possibly due to promotion of the Saskatchewan Immigrant Nominee Program. Over 50% of recent immigrants to Saskatchewan, Manitoba, Prince Edward Island, New Brunswick, and Yukon were admitted under provincial nominee programs, while nationally only 16.4% of recent immigrants were admitted under such programs [8]. However, current provincial health system strategic plans do not mention any initiatives that directly respond to the healthcare needs of newcomers (immigrants and refugees) [9].

Newcomers to Saskatchewan may experience a variety of barriers to accessing healthcare services, including low health literacy, lack of familiarity with the healthcare system, cultural competency, cost, traditional beliefs, language difficulties, appointment time limitations, and lack of childcare or transportation [10]. Refugees may also experience additional barriers to healthcare due to discrimination and stigmatization [11]. Since newcomers’ ability to access healthcare can be impacted by cultural, religious, linguistic, and health status differences, healthcare systems need to be designed to equitably accommodate newcomers [12]. A variety of options are available to support newcomer health, including the use of basic English and other languages in public health information, engagement with immigrant communities to advise on program development, offering culturally competent health services, interpretation services, and through creating space to collaborate with traditional practitioners [13,14]. Many of the inter-related initiatives that may support newcomer health appear to revolve around the provision of culturally competent health services.

The Western biomedical model has been described as invalidating immigrant spiritual and religious beliefs, familial context and their understanding of health through universalizing the human experience; and is subsequently an ineffective approach to culturally sensitive care [15]. Culturally competent care has been defined as “a dynamic, fluid, continuous process whereby an individual, system, or health care agency finds meaningful and useful care delivery strategies based on knowledge of the cultural heritage, beliefs, attitudes, and behaviours of those to whom they render care” [16]. Moving towards culturally competent care entails a critical examination of institutional policies and procedures to identify how they may be contributing to the disempowerment of vulnerable groups [16]. Culturally competent healthcare services have the potential to increase health system efficiency and client satisfaction, and improve newcomer health outcomes [17], as well as build a link between traditional beliefs and practices with those of Western medicine [18]. The overall objective would be to bridge diverse cultural perspectives with Western medicine to facilitate better understanding on both sides without forcing anyone to supplant their beliefs with new ones. 

Several studies have focused on the evaluation of healthcare services that target immigrant women. Immigrant women in Toronto perceived that a mobile health clinic met their care needs by providing highly accessible, holistic, and culturally and linguistically appropriate healthcare services [19]. Health promoters have reported that creating relationships with their clients was key to successful health promotion [20]. They described the importance of being culturally responsive, establishing trust, demonstrating respect, and engaging in reciprocal learning with their clients to successfully engage them in health promotion activities. These studies demonstrate that targeted innovative programming can be developed to support immigrant women’s access to healthcare. 

The community health centre (CHC) model in Toronto appears to successfully reach immigrant populations. In a study of self-managed care and health service use in Toronto, Hyman et al. [21] observed that Black Caribbean immigrant participants were more likely to access care at the CHCs and receive diabetes care from a nurse educator and were more likely to engage in positive diabetes self-management practices, including regular A1C testing, as compared to the Canadian-born. The CHC health care model appears to be a promising best practice for the provision of health care to immigrants with chronic conditions. 

Existing research provides a wealth of information on the barriers and recommended supports to improve newcomer healthcare access. However, most intervention studies targeted one specific ethnic group, often in larger metropolitan centers with a longer history of minority immigration, as compared to the relatively recent influx of immigrants to Saskatchewan. Although immigration is increasing in Saskatchewan, the healthcare system has not yet prioritized service adjustments to respond to immigrant healthcare needs [9]. Recent research has demonstrated that Saskatchewan newcomers are experiencing substantial barriers to healthcare [10]. This article explores newcomer families’ experiences with healthcare during their first 5 years in urban areas of Saskatchewan, Canada and their suggestions for healthcare improvements with the aim of informing the development of an accessible newcomer healthcare model. 

## 2. Materials and Methods

This study is part of the larger multi-method Healthy Immigrant Children study that encompasses a comprehensive assessment of 300 newcomer children’s health status and access to healthcare [22]. This methods section describes the qualitative methods used for this article. 

### 2.1. Participants

Several newcomer settlement organizations, such as Open Door, facilitated participant recruitment by inviting newcomer families to participate. Participants were families with children aged 3 to 13 years living in Regina or Saskatoon and had been in Canada for less than 5 years. Ethical approval was granted by the University of Saskatchewan Research Ethics Board. The first author obtained consent and conducted all interviews in English or Spanish during 2015. An interpreter was required on one occasion, while other participants declined the offer. 

We planned to interview between 15 to 20 parents to reach data saturation, which can usually be reached with 8 to 12 interviews [23]. Efforts were made to purposefully select newcomer families from immigrants and refugees involved in the larger study to approximate the typical ethnic origins of recent newcomers to Saskatchewan. However, due to frequent moves we could not contact many of the Asian refugees to invite their participation in this part of the study. In 2016, 68% of Saskatchewan newcomers were from Asia, 13% from Africa and the Middle East, 14% from Europe, 3% from the Americas, and 2% from the United States [24]. Twenty-two parents from 19 distinct newcomer families participated. Parents were not asked to provide their ages. Both parents in three families participated as couples. Participating parents included 15 mothers and 7 fathers, and 13 immigrants and 9 refugees. Participants originated from Eastern Europe (1), Latin America (1), Africa (2), the United States (2), Western Europe (2), Asia (6), and the Middle East (8). American and Western European families reported high incomes; one Middle Eastern and one Latin American family reported middle incomes, while the remainder reported low incomes.

In addition, we reached out to all known refugee settlement and immigrant serving organizations, English-as-a-second language programs, schools with high immigrant populations, and healthcare providers at clinics that served large immigrant populations in Regina and Saskatoon. This outreach resulted in 22 service providers consenting to participate in similarly structured in-depth interviews about their concept of optimal newcomer healthcare services. Service providers did not provide demographic information.

### 2.2. Theoretical Orientation

The concept of ‘candidacy’ as described by Dixon-Woods et al. [25] provides a useful conceptual framework to understand healthcare access. Candidacy encompasses how an individual’s eligibility for healthcare is determined through a process of negotiation between themselves and healthcare services. According to Dixon-Woods et al. [25], candidacy is “a dynamic and contingent process, constantly being defined and redefined through interactions between individuals and professionals.” Healthcare access is determined by the interaction between macro-level healthcare structures, including service configuration and resource allocation, and an individual’s capacity to attain services, which may be facilitated or constrained by their social context. Healthcare services usually have eligibility criteria and established processes for accessing services, as well as criteria for prioritizing those who require urgent medical attention. Prior to accessing healthcare, individuals decide when it is appropriate to access healthcare, which can be either supported or deterred by their socio-economic status and their perceptions of healthcare services expectations, care environment, and health status. When there is a lack of alignment between healthcare service structures and an individual’s embodied capacity to cross the access threshold, vulnerabilities can be created among disadvantaged people.

### 2.3. Data Collection and Analysis

In-depth interviews were scheduled with parents; however, sometimes extended family members joined the conversation and consented to participate, resulting in household interviews. Interviews were transcribed verbatim, with the speaker noted whenever possible, and the entire data set was analyzed. The in-depth interview guides included questions about their experiences accessing care in the Canadian healthcare system and suggestions for improvement (Table 1). Existing questionnaires were accessed to guide the development of interview questions [13,26,27]. Open-ended questions were used to collect rich, descriptive narratives [28]. Interviews were conducted until data saturation was reached and further interviews did not yield new information beyond that already collected.

Among services providers, sometimes two or three staff members from one organization were interviewed as a small group, and interviews were transcribed with speakers noted whenever possible. The interview guide included open-ended questions modified from the parent interview guide, as well as additional questions that explored current understandings of factors that may be impacting access to healthcare. Consistent with the newcomer parents’ interview process, interviews continued until saturation was attained. 

The theoretical lens of candidacy provided a framework that guided the initial thematic content analysis of the interview data. Interviews were recorded and transcribed verbatim, and then verified a second time by the same person. Data were initially coded using an inductive approach to generate categories [29]. Salient data extracts related to accessible healthcare were identified and used to generate coding categories. Initial categories were reviewed to identify main themes with reference to the frequency of similarly coded data extracts and relevant atypical experiences. Categories were combined and reorganized to further refine themes. Data analysis was initiated shortly after interviews started in an iterative process, whereby initial results suggested additional lines of investigation, which resulted in more nuanced questions and probing during the later interviews. A second researcher reviewed the coding framework with reference to the initial five interviews. NVivo11 was used to organize and code the data.

Interviewing to the point of data saturation, expansive probing, and prolonged participant engagement over several study components supported achieving thick description [30]. In addition, service providers drew on their broad experience with many newcomers, theoretically increasing the sample size underlying the data set. The inclusion of both parents and service providers allowed for data triangulation from these two data sources. Asking about both positive and negative experiences with healthcare and using an initial inductive approach to data analysis supported reducing researcher bias. 

Data reliability was supported through an iterative interview and data coding process. As interviews proceeded, probing expanded to inquire about new lines of investigation, and the context of knowledge gained from earlier interviews influenced coding decisions [30]. Data reliability in the Saskatchewan context was supported by purposefully selecting an ethnically and socioeconomically representative sample of recent newcomers. Internal reliability was evident in the overlapping interview data from distinct participants.

Quotes are attributed to participants according to the following naming conventions: Regina refugee (RR), Regina immigrant (RI), Regina service provider (RSP), Saskatoon refugee (SR), Saskatoon immigrant (SI), and Saskatoon service provider (SSP). 

## 3. Results

**Figure 1:**
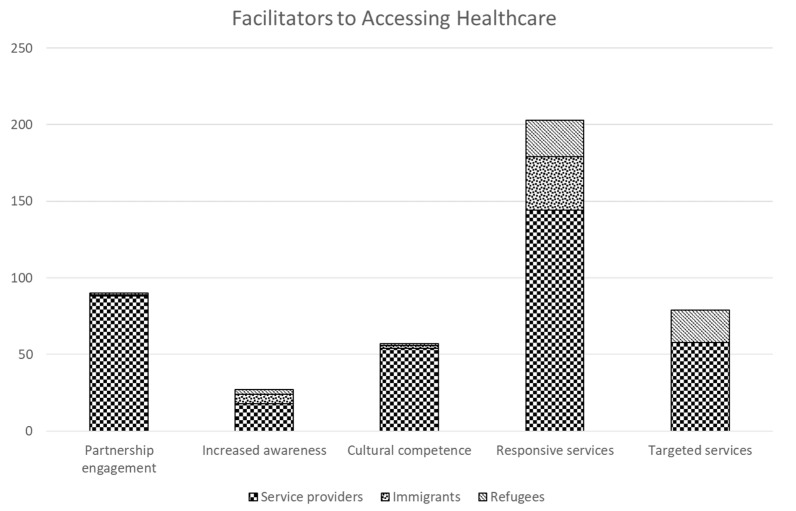
Frequency of data extracts coded to themes by interviewee type.

Analysis of participant quotes related to accessible healthcare services revealed 5 main themes: (1) responsive, accessible services, (2) increasing cultural competence, (3) targeted newcomer health services, (4) increasing awareness of health services, and (5) newcomer engagement in planning and partnerships. Throughout the results section priority is given to including newcomer quotes when available; however, service providers often had more direct knowledge of how the healthcare system works and opportunities for improvement. The frequency of participant statements related to each theme is outlined in Figure 1. Responsive services was the most frequently coded theme among all participant groups. Refugees and service providers contributed to the targeted services theme. The perspectives of service providers largely shaped the partnership engagement and cultural competence themes.

### 3.1. Theme 1: Responsive, Accessible Services

Both service providers and newcomers recognized the importance of responsive healthcare services, including convenient access, as core components to build an accessible healthcare system. Newcomers mainly spoke about the importance of convenient access to healthcare services, while service providers suggested the development of integrated primary healthcare sites, not exclusively for newcomers, but with comprehensive services provided in a culturally responsive manner.

In order to access publicly funded healthcare, newcomers need a Saskatchewan health card. Unlike several other provinces, Saskatchewan deems all new permanent residents eligible for a health card immediately upon arrival. When the Ministry of Health became concerned that immigrants in other provinces were coming to Saskatchewan to get a health card that they could then use until they were eligible for health coverage in their home province, they consulted with Regina Open Door about how to ensure that only permanent residents who intended to reside in Saskatchewan could get a health card. The Ministry of Health responded to concerns about their policy that required a permanent address and was able to work out a process that allowed for immediate access to health cards for legitimate newcomers to Saskatchewan.

*…newcomers would come to Saskatchewan to get a Saskatchewan health card right away and then use it for 3 months in BC or Alberta…they cracked down on this by saying give me your permanent address when you apply, but refugees…don’t get into permanent housing right away, they stay in temporary housing…we said what if you provide a temporary card for 1 month and we will sign to let you know the person is here…and after 1 month we send you the address and they send them the full health card*.

(RSP1)

Many healthcare providers shared ideas for more accessible healthcare services that respond to the needs of vulnerable populations, including newcomers, such as conveniently located healthcare services with streamlined access. In general, healthcare providers suggested that a one-stop shop with co-located services and readily accessible would be ideal.

*A lot of these folks cannot make appointments; they don’t know what life is going to look like so drop in clinics work well…the health bus 1 day a week…is parked in a neighbourhood that is very close to where a lot of these folks live…and it’s on a Saturday …Monday to Friday doesn’t work for them; they want weekend appointments*.

(SSP1)

Newcomer participants placed a high value on convenient healthcare services. Once newcomers became aware of walk-in clinics, they found them very convenient for meeting a variety of health needs. Conveniently located clinics that do not require appointments and are open longer hours were preferred by many newcomers to overcome some barriers, such as transportation, limited opportunity for daytime appointments and language difficulties.

*…the walk-in clinic is better for health services because newcomers don’t always speak English…for some speaking is hard. With walk-in clinic you don’t need to phone for appointment*.

(SR1)

Responding to newcomer healthcare needs often means reflecting on your normal process and adjusting it as necessary to better accommodate newcomers. Many healthcare providers went the extra mile to provide support needed to ensure children can access programming and women are provided with urgent care in a respectful manner.

*…offered cab vouchers…tried to arrange group bookings…so they are not having to come for another appointment…hired some interpreters that are on site…(and) email the interpreter to contact the (client)*.

(RSP2)

In addition, sometimes extra time needs to be dedicated to building relationships with newcomers, so they feel comfortable accessing services.

*…any of these mandates that we are trying to…be efficient and not waste money, that doesn’t necessarily work in these scenarios. You have to put the human/individual first and the relationship develops…once you have that relationship going the people are open and you can work better…but that takes time*.

(SSP1)

Interpretation is a vital component of responsive healthcare services for newcomers with limited English. Immigrant service providers can be the link to arranging interpretation services and serve as the contact point for referrals from English speaking healthcare providers.

*The referral needs to be coordinated. All the clients…for 2 to 3 years still have a language barrier and work through us. I am primarily the person booking an appointment or making a referral to family physician and…dental or eye appointments…they always put (us) as…primary contact to coordinate the appointment with the specialist*.

(RSP3)

Service providers emphasized the importance of consistent access to convenient high-quality interpretation services during healthcare provision. In addition, some resourceful newcomers sought out responsive healthcare providers to meet their needs.

*I used to book him (husband) with some Indian doctors so that he could go and visit them and talk to them, rather than me interpreting for him…(he can) talk to them so that…he could deal with his own situation…like I am not (such a) dependent person*.

(RR1)

Public health services were identified as an important responsive service that does outreach to newcomer families, often in conjunction with schools, to help connect them to healthcare services. When asked about which healthcare services they had accessed for their children, many newcomer parents mentioned public health services. Aside from the school and church clinics, Saskatoon public health services appear do active outreach. Regina newcomers also mentioned accessing public health services; however, there did not seem to be the same level of organized outreach to the newcomer population.

*The public health nurse came here several times, once she came here (current house) and so many times she came to Stonebridge (previous residence) in the first couple of months*.

(SI1)

Healthcare providers emphasized the importance of convenience and outreach in the provision of public health services to support the health of newcomers.

*A lot of the newcomers settle in an area that has a school very close…and they have a drop-in immunization clinic every Thursday so a lot of those folks access that service simply because of the geographical location and then word of mouth*.

(SSP2)

In addition, Saskatoon public health services either provide or connect newcomer families to low-cost dental care. Although public health services are providing high quality services, participant comments indicate opportunities to review services with a critical focus on the provision of outreach and comprehensive services to support newcomers during their first few years in Canada.

As Saskatchewan’s newcomer population continues to expand, it will be necessary for healthcare organizations to reflect on their capacity to recognize and understand the needs of newcomers and to adjust their practices to respond accordingly. An evaluation of current healthcare services with a cultural lens will assist with planning the way forward to meet the healthcare needs of a diverse population.

*…how prepared are we and what kind of skills do we have in actually meeting the needs of people…making sure that staff know…how they can access (interpretation) services and creating a culture where that is the first approach, rather than just deferring to I don’t know how to do that…developing policies that actually ingrain this idea into their work culture of how do we support our newcomers*.

(SSP2)

Many service providers were aware of supportive healthcare models used in other cities that incorporated interpretation services, cultural brokers, and health facilitators. The development of integrated primary healthcare sites that provide comprehensive services with convenient access in a culturally responsive manner that incorporates the role of cultural facilitators would provide ideal service for many newcomers.

*…the continuation of care is important so if the health system in Saskatchewan has these primary care centres…they are open for the newcomers and take care of them…a place where they can see their family physician, their own psychologist or counselor, nutrition therapist…A multidisciplinary clinic*.

(RSP4)

The use of mentors or host families to provide additional guidance on an as-needed basis during the first few months in Canada was mentioned by many service providers and newcomers as an ideal model to support newcomer access to a variety of services. Healthcare providers recognized that some newcomer families struggle with accessing healthcare and emphasized the primacy of mentorship and the role of the public health nurse in collaborating with formal and informal mentors. Although many immigrant-serving agencies offer host family programs, perhaps there could be additional training for host families to encourage their involvement, and/or healthcare providers could be made aware of these programs and be open to collaboration with the client and their mentor when appropriate.

*That support is missing sometimes…who is there that could help that particular family to make sure they access that service? As a public health nurse, it’s helping to identify who their mentors are, if they have a church member or community member to help them, talk together to make sure they are able to access resources*.

(RSP5)

When asked about policy suggestions to improve newcomer health, healthcare providers recommended improving health benefit programs to make prescription drugs and dental and vision care more accessible, as well as universal childcare so parents can more easily integrate into employment. Immigrant service providers suggested more prevention programs designed with a newcomer lens, as well as gender and Aboriginal lenses. In addition, immigrant service providers mentioned some specific policy challenges that make it difficult to support newcomer integration. Many government services prefer to provide service over the phone instead of in-person drop-in service, which presents significant challenges for newcomers with limited English skills. Current privacy guidelines have not been designed through a newcomer lens and can be a barrier when a caseworker is trying to assist a newcomer client to access a government service. For example, the reporting system for the housing supplement relied heavily on telephone contact, which is a barrier for newcomers.

*…they say ‘no I cannot share with you because there is a privacy policy.’ I say ‘but I have the newcomer here, I have a signed document here,’ ‘no let the newcomer talk to me,’ but he can’t talk because he doesn’t speak English…A key point is creating a circle of care team with the organizations working with newcomers…and other service providers…in terms of how we can share and protect information*.

(RSP1)

These examples speak to the importance of applying a newcomer lens to all programs and policies to ensure equitable access without creating an administrative workload burden for immigrant settlement agencies. 

### 3.2. Theme 2: Increase Cultural Competence

Although not commonly mentioned by newcomers, service providers often agreed that increasing the cultural competence of our healthcare system would contribute to the development of accessible healthcare services for newcomers. They noted that service providers are often unaware of cultural practices and traditional medicine in different countries and how these practices impact access to healthcare.

*…there are still people…who have no idea what Islam is or Ramadan is…there is a real knowledge gap there so it would probably be helpful to know what is appropriate and what’s not. Trying to understand where people are coming from a little better before asking certain questions*.

(SSP3)

However, there is a balance that must be achieved between attenuating the healthcare system to meet the needs of newcomers and educating newcomers on how the Canadian healthcare system works to support the development of a shared understanding.

*…we also have to somehow bridge that gap, we are in Canada and people are coming here and they are learning…how things operate here…there is a balance…there is that meeting place where we both come together*.

(SSP2)

The need to provide culturally sensitive dietary advice and education was highlighted by service providers. Health education messages need to be customized to be relevant to people from different cultures and respectful of their traditional knowledge. The current Canadian Food Guide was designed from the viewpoint of those who consume a Western diet, so it often fails to be an adequate dietary guide for cultures whose staple foods include maize or pulses.

*What’s really important is…not having that expectation that the way we eat or the foods we have are the foods that our newcomers should be eating…how can you translate good nutritional practices into the dietary practices that they bring with them*.

(SSP1)

In addition, newcomers may not view organized physical activity as a recreational activity if they have done physical work all of their lives and walked everywhere. Hence, they may not recognize the importance of getting their children to be physically active. Adopting the newcomer’s perspective before delivering health education messages can lead to more successful health promotion among newcomers.

*…explore what it is within that culture, their beliefs around breastfeeding, beliefs around being active…how can we blend it to (Canadian recommendations)…in the long run it would be so powerful*.

(RSP5)

Several newcomers mentioned the use of traditional healing practices to prevent illness and as a first-line treatment before seeking medical help. It is not clear if any of them have ever mentioned their traditional healing practices to their healthcare providers or if they would be comfortable doing so.

*I give them warm milk with garlic for coughing and it is better…runny nose then it is gone. Sometimes it helps and we don’t need to go to doctor. Sometimes it doesn’t help and we go to the family doctor or clinic*.

(SR2)

Some service providers mentioned that increasing understanding of traditional and alternative healing practices among healthcare providers would contribute to a supportive healthcare environment. Service providers noted the importance of respecting traditional health beliefs, which can be core cultural components of some newcomers’ identities while providing evidence-based health advice. It is also important to be aware of any traditional treatments being used by patients to ensure they are not contraindicated for the client’s condition.

*…the healing forces by holy water, the prayer, and also when the community are together and strong to support the family that is psychological healing…at the time of sickness the whole community stands with you…In childbirth in Eritrean culture…the mother will have traditional sauna…and when she comes out the flaxseed soup…she have to drink…it makes her muscles loose so it helps her at delivery time*.

(SSP4)

### 3.3. Theme 3: Targeted Newcomer Health Services

The Regina Open Door Society, former Regina Qu’Appelle Health Region and Regina Community Clinic have partnered to offer targeted health services to refugees in the Regina area since 2004. One of the keys to success has been the availability of supportive interpretation services. The overall success of the Regina partnership in terms of healthcare access measures and client satisfaction is high.

*…(the partnership) has been working very effectively…resulted in coordinated healthcare for all individuals with high medical needs (and) … 99 to 100% success in terms of immunization because we do close follow up…Also in terms of going back to medical clinic, visiting the family physician is significantly high at 98 to 99%*.

(RSP2)

In some instances, quick access to the targeted healthcare service has been beneficial for those with existing health issues, such as a newcomer who had a potentially stigmatizing mental health issue and had facilitated access to good treatment that assisted him to recover and successfully integrate into Canadian society.

*…we were supported by Open Door that was the good thing, we were booked by the public health nurse to see the doctor…My husband was on medication that was why we got into the doctor because he used to get his medication for the depression…After he got the job and started working and he is good now. He doesn’t need it anymore. He got good care from Regina Community Clinic*.

(RR1)

The Regina Community Clinic is meeting the healthcare needs of refugees, although they are experiencing increased service demands such that they are stretched to accommodate with existing resources. The Regina Community Clinic accepts all new government-sponsored refugees to the Regina area as patients, and when many of them do not transition to other physicians in the community, it can become difficult to continue the same level of service without the addition of new resources.

*When we started, we started with 20 or 30 patients a year…and then the number increased every single year, now…we take 200 to 250 patients a year…90 or 92% of them stayed in the clinic since they started…even when we tell them they can go find another physician in the community they don’t…they like the clinic, they come for follow ups, if they have a translator and somebody to bring them they don’t miss their appointments*.

(RSP4)

Even demand for the public health services provided by the former Regina Qu’Appelle Health Region at the Regina Open Door Society location is growing.

*Every year we do flu clinic for…this year close to 700 (individuals)…for us 700 is huge number…(public health nurse) sees more people, sometimes we have more people waiting in that section than the other*.

(RSP2)

Although the former Regina Qu’Appelle Health Region Public Health Services and Regina Community Clinic are providing targeted service to refugees, immigrants do not have access to the same dedicated service. Given this lack of targeted services for immigrants and the increasing demand for supportive healthcare services by refugees, some service providers suggest the need for a primary healthcare clinic that focuses on newcomer health.

*Our health program is mainly for refugees…we have proposed to have a primary health site that focuses on newcomers…Lack of interpretive services is one of the reasons people are not going (to other healthcare services). Immigrants are not getting interpretive services, even refugees are only getting the service…for the first 6 months while they are going through the health partnership*.

(RSP1)

In Saskatoon, a nurse practitioner at Westwinds Primary Health Clinic sees many refugees who are referred to her by the Saskatoon Open Door Society. Typically, she receives requests from settlement counsellors when they have new arrivals that need a health exam, and an appointment is made with consideration given to the urgency of the referral. Sometimes settlement counsellors may think a referral is urgent when it is not, possibly because they do not have access to a dedicated public health nurse to do the timely screening. Although in-person interpretation services are not routinely provided for clients who see the nurse practitioner, she has access to telephone interpretation services through the former Saskatoon Health Region’s account when needed.

*(nurse practitioner)…makes sure their immediate needs are met anticipating any sort of issue or problem, trying to address it for them, giving them some ideas about where they can go…just letting them know if they have a question or are not sure about where to go …(they) can ask…(she is) a facilitator and points people in the right direction… sometimes (she sees refugees) over a long period of time simply because they don’t want to see anybody else*.

(SSP2)

As previously discussed, rather than developing targeted services for newcomers, the former Saskatoon Health Region has focused on expanding programming to reach vulnerable populations, including newcomers, who live in low-income areas. An exception to this approach is in the area of mental health, where the former Saskatoon Health Region partnered with an immigrant-serving agency to offer counselling on-site to overcome some access barriers.

*…we have a counselor that comes Tuesday afternoons…employed by the health region…people can bypass the whole intake process to come in and see a counselor here. There is definitely people who would not come to see her (at the health region site)…There is a lot of stigma with mental health*.

(SSP5)

Similar to Regina, there is not a standard referral system for immigrants to access dedicated healthcare providers in Saskatoon. A coalition of healthcare and immigrant service providers was collaborating on the Providing Access to Healthcare (PATH) project to develop standard processes in the healthcare system to meet newcomers’ healthcare needs in Saskatoon.

*The intention of PATH is trying to create pathways and processes between healthcare providers, where newcomers can arrive in the city and there would be established processes…that people would move through the system, so strong connection and communication…methods of referral, and practitioners who are on board to be able to really wrap around the diverse needs of the newcomers*.

(SSP6)

In addition, some service providers advocated for a newcomer primary healthcare clinic to serve refugees, immigrants or both populations.

*…anything that helps to build that trust…it is a mutual effort, cultural competency of the staff and health care providers, as well as the reliability of the services and the connection that the patient can feel with the doctor….a community clinic…that can provide services directly to immigrants and refugees would help them to open up easier*.

(SSP5)

### 3.4. Theme 4: Increase Awareness of Health Services

One of the key prerequisites to accessing healthcare is being aware of the services and how to access them. Newcomers may get information from a variety of places including immigrant settlement agencies, newcomer welcome centres, public health nurses and their neighbours. Therefore, they do not always get accurate or timely advice when they have a need for healthcare service. 

Several newcomers, more commonly immigrants, mentioned difficulties with not knowing where to access healthcare and that orientation to healthcare services would be helpful. Although refugees have access to caseworkers at immigrant settlement agencies, immigrants do not get the same level of service, which may put them at a disadvantage.

*Better orientation would be good. We didn’t have any of our family members or relatives or any family friends when we came to Regina, we had no contacts, it takes time to know your community centres*.

(RI1)

However, English-speaking immigrants appeared to understand where to look for information on community resources.

*Open Door Welcome Centre told us about healthcare here…they gave us a brochure and health card application…I think there are things on the government website/health site, to say what you can expect coming into the country*.

(RI2)

Service providers generally agreed that we need to systematically inform newcomers about the healthcare system so they are better prepared to access healthcare services when the need arises. They recognized the need for multiple modalities to assist newcomers to understand and connect to the healthcare system. In addition, some newcomers with more complex healthcare needs may require more intensive short-term case management type services to assist them to connect with healthcare services.

*…hospitals…(could) be open to tours…an orientation to how a hospital works (and providing) more knowledge before they come, pre-arrival information, that seems to be a huge issue for a lot of people, a lot of assumptions being made, what Canada is, the kind of services*.

(SSP4)

### 3.5. Theme 5: Engagement in Planning, Partnerships

The importance of partnerships in the development of health programming for newcomers was stressed by many service providers when they spoke about existing successful programs or those that are currently under development. Successful partnership development requires ongoing communication and senior leadership involvement. Service providers mentioned several examples of successful partnerships, including partnerships with MEND (Mind, Exercise, Nutrition, and Do It) to offer health promotion programming to youth and a local charity to support the health and social needs of newcomer youth that are not covered by other programs.

*MEND (Mind, Exercise, Nutrition and Do It)…A University of Saskatchewan program…One of my groups…I sent them to MEND…they talk about healthy eating, being active, screen time. One session of MEND is only for Open Door clients…It is at YMCA…our funding is for transportation…It is a good program*.

(SSP4)

Many organizations recognize that everyone holds a piece of the partnership puzzle and cannot succeed alone, so there is a need to communicate with each other to develop successful collaborative health programs. Some barriers to collaboration that need to be overcome include overly narrow funding parameters and established ways of working that need to be worked through.

*Somebody may have a program already out there that could be beneficial for newcomers, but…they say ‘no, we are asked to focus on this particular population and don’t have the funds to work with yours.’ We need more flexibility to work together*.

(RSP1)

In addition, sometimes partnership building work is not prioritized amongst the daily service delivery demands. In Saskatoon, service providers were in the initial stages of building the PATH partnership and realized that communication is key to moving forward.

*…right now we work quite separately…settlement does this and (health system) does that, but if we build more pathways between and amongst our services there would be more support in trying to develop pathways for newcomers to access services*.

(SSP5)

There was strong support among service providers to consult with newcomers about healthcare planning to ensure that their healthcare needs were considered. Service providers mentioned consultation gaps and lack of responsiveness to suggestions, as well as opportunities to consult with newcomers.

*…they need to be at the decision-making table…provide that wisdom and guidance…just as much as it’s really big with our First Nations and Metis. You can’t make decisions for people or on behalf of people, they need to be invited to the conversation at the table because what they have to say is important. They have the understanding*.

(SSP7)

One service provider mentioned that she was surprised to be consulted by the federal corrections service when she had not been invited to provide cultural advice to the healthcare system.

*I was very disappointed when (Healthline) 811 was launched, nobody contacted us, they didn’t have any strategy to promote it among newcomers, although it is an excellent and valuable service…We don’t get opportunities in some areas…when developing policies. Like primary health care for example, I haven’t heard from anybody*.

(RSP1)

When meaningful consultation happens, it can have a substantial impact. However, healthcare planners must remember to thoughtfully include the growing newcomer population in their demographic overview to inform healthcare consultation plans. Service providers’ comments conveyed that successful consultations were often facilitated by healthcare administrators becoming aware of someone they could connect with from an immigrant-serving organization.

*…for years they didn’t include anything on newcomers…I phoned someone and said there is nothing on newcomers and they said they forgot. How can you forget this when people are coming here in droves?…now there has been consultation. …the creation of the Saskatchewan Mental Health Coalition and the invitation to Open Door to be part of that forum and have a voice at that table and also having a component on immigrant and refugee health being put into their report…not just the First Nations and Aboriginal populations*.

(RSP1)

Beyond an advisory capacity, service providers also offered some suggestions about how to engage newcomers in leadership roles.

*…there is a lot of capacity for internationally trained health professionals who have a lot of valuable input and time to be used…(they could assist with) some expertise in terms of health literacy*.

(SSP5)

**Figure 2:**
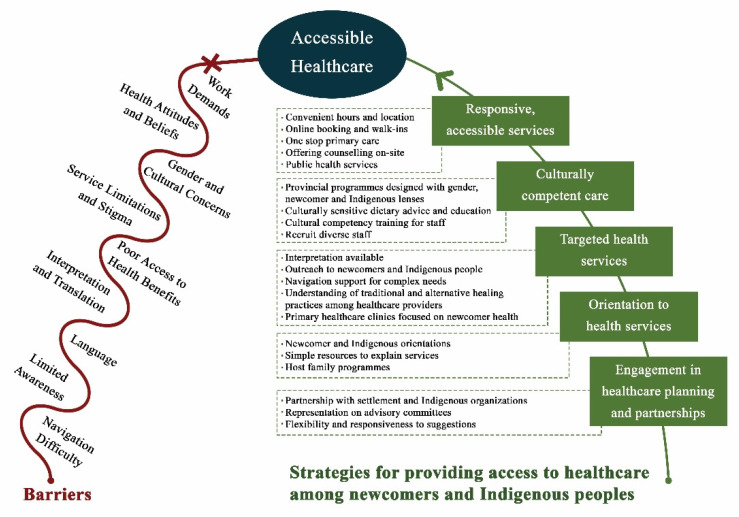
Strategies to support accessible healthcare.

Strategies to support accessible healthcare are summarized in Figure 2.

## 4. Discussion

In alignment with best practice recommendations regarding healthcare access [12], participants recommended that healthcare systems should be designed to accommodate cultural, religious, linguistic, and health status differences common to newcomers. Participant comments indicate that the provision of culturally competent health services may be at the center of all the inter-related initiatives that support newcomer health. It is the link between the core identities of newcomer groups to Westernized medical care. Since the Western biomedical model has been found to be an ineffective approach to culturally sensitive care [15], efforts should be made to move towards culturally competent care through a critical examination of institutional policies and procedures to identify how they may be contributing to the disempowerment of vulnerable groups [16]. Culturally competent healthcare services can bridge traditional beliefs and practices with those of Western medicine [18] and has the potential to improve newcomer health outcomes and client satisfaction [17]. The overall objective should be to facilitate better understanding between Western healthcare providers and cultural groups without forcing anyone to supplant their beliefs with new ones.

In alignment with participant suggestions, the use of culturally and linguistically appropriate health education materials can produce positive results, such as increased HIV testing [31] and increased client satisfaction [32]. Overall, it would be beneficial to enhance the cultural capacity of the healthcare system to support newcomers to feel respected and welcomed, as well as to ensure that treatment recommendations consider traditional beliefs and practices to support the best clinical outcomes and client satisfaction. 

The first step to accessing healthcare services is to understand where and how to access services, so a systematic approach to providing this information to newcomers would help to facilitate access. Consistent with participant comments, previous research has demonstrated that recent newcomers often lack understanding of how or where to access healthcare services [33]. Participants noted that settlement agencies and ethno-cultural organizations/networks are reliable sources for this type of information, but newcomers, especially immigrants, who do not engage with these organizations may not encounter this information. There are also other opportunities to enhance awareness of healthcare services, including the provision of pre-arrival information, hospital tours and outreach services from Public Health.

All participating healthcare and immigrant service providers agreed that there is a need to make adjustments to the healthcare system to more systematically address the health needs of newcomers. However, perspectives ranged from providing supports to assist newcomers to better access universal healthcare services to the development of specialized primary healthcare sites for newcomers or refugees. Consideration will need to be given to whether a targeted or inclusive approach to healthcare is adopted. Several studies have found that both targeted newcomer programs and more universal community health centers can successfully serve the newcomer population. For example, a mobile health clinic that provided primarily reproductive healthcare services to immigrant women demonstrated some positive results with regards to providing accessible, holistic, and culturally and linguistically appropriate healthcare service [19]. Similarly, the community health center model (CHC) successfully engaged with Black Caribbean immigrants such that they were more likely to access diabetes care from a nurse educator than the Canadian-born [21]. 

While the development of targeted primary healthcare sites that provide culturally responsive services may be ideal for many newcomers, targeted programs can quickly develop capacity issues if clients do not transition to regular universal services after the first year. Depending on eligibility criteria, targeted programming may be inaccessible to some immigrants who need similar services. The other option is the inclusive approach, whereby a newcomer lens is adopted to guide the transformation of healthcare services. In low-income areas, this may include the development of inclusive healthcare services for all vulnerable populations, which incorporates culturally sensitive care for both First Nations and newcomer populations, including interpretation. Consideration may also be given to hiring some cultural facilitators to assist newcomers with intensive or complex health needs to access healthcare services at various facilities. 

Service provider comments emphasized the importance of good communication between organizations and a willingness to share resources and expertise to develop successful healthcare partnerships to serve newcomers. Effective partnership building requires focused work by all involved organizations to come to an understanding about how everyone can contribute to building a collaborative healthcare model for newcomers. Service providers mentioned several partnerships that are key to ensuring program success, either through engaging participants to use the service or providing supports such as transportation or facility use. However, sometimes overly narrow funding parameters or established ways of working together can be barriers to partnership development. Research has demonstrated that successful healthcare partnerships entail meaningful consultation and involvement with the newcomer community [14]. When community members are consulted as experts and treated as true partners in a collaborative program development process, the focus is placed on cultural relevance, and long-standing problems can be resolved [30]. Newcomer communities and settlement organizations can be valuable partners in the development of responsive healthcare services.

The identified themes generally align with the concept of ‘candidacy’ described by Dixon-Woods et al. [25]. The identified themes focus on the interaction between healthcare services, including service configuration and resource allocation, and the social context of newcomers’ lives, which together impact capacity to access healthcare services. Overall, newcomer healthcare services should be designed in collaboration with newcomers in a systematic and reflective manner to respond to the cultural, social and economic contexts of newcomers’ lives; and healthcare orientation should be proactively offered to newcomers to support appropriate and effective access to available services. This alignment of responsive healthcare services with an enhanced understanding of how to access care can reduce inequitable healthcare access among newcomers.

The Saskatchewan health system has made some policy and practice advances related to serving newcomers over the past few years. The former Saskatoon Health Region released a reference guide for healthcare providers in their region to access interpretation and translation services in 2013 [34]. The guide details how to access a telephone interpreter, health region staff or volunteer interpreter, and sign language interpreter, but leaves it to the reader to decide which type of interpretation is most appropriate in each case. The former Regina Qu’Appelle Health Region has provided information to their staff on how to access Can Talk telephone interpretation and services for those who are deaf and hard of hearing. In mid-2014, they also informed all staff that they should use Can Talk service prior to seeking out a staff member who translates [35]. This may have been in response to concerns about client confidentiality or interpretation accuracy issues. However, Can Talk has not worked well for everyone, and face-to-face interpretation is required for some patients [35]. 

The former Saskatoon Health Region is also providing leadership related to supporting internationally trained healthcare workers to engage in employment in Saskatchewan. In collaboration with the Ministry of Health, and with funding from Health Canada, the Saskatoon Health Region leads a provincial project, Pathways: Internationally Educated Health Professionals (IEHP) Support, Bridging and Integration, which provides information and support to assist IEHPs to achieve licensure and seek employment within their professions [36]. Although the primary motives for undertaking these projects may be to support human resource needs, the projects will likely assist with transforming the healthcare system workforce to more closely resemble the population served in Saskatchewan and perhaps becoming more open to considering how to meet the needs of the newcomer population.

The inclusion of diverse newcomer groups in terms of culture, country of origin and socioeconomic background, as well as service providers, is a strength of the study that supported integration of diverse perspectives of both consumers and service providers regarding how to improve healthcare. The study results offer insights on how to improve healthcare in Saskatchewan and similar contexts to improve newcomer health outcomes. Unfortunately, we were not able to reach sufficient Asian refugees due to frequent moves by these families, resulting in lower than desired participation by this group. In addition, study recruitment was limited to urban centres, so the experience of newcomers in rural and remote areas that may have experienced different or more intense challenges is not included. Service providers often contributed more complex ideas grounded in their many years of experience within the healthcare and immigrant-serving organization environments and supported by their advanced English language skills, resulting in the inclusion of many service provider quotes. However, whenever possible relevant newcomer quotes were prioritized for inclusion.

These study results and similar literature suggest that future research should focus on trials of healthcare models that offer services targeted to newcomer families as compared to more universal healthcare models that integrate responsive, culturally sensitive care practices. Such trials could help point out which model results in the best outcomes, as well as determine whether either service model can better meet service demands within available resources. 

## 5. Conclusions

An accessible healthcare system should include primary healthcare sites developed in partnership with newcomer service organizations to offer comprehensive care in a conveniently accessible and culturally responsive manner, with embedded interpretation services. Since specialized programs for a target population may unintentionally isolate clients from the broader healthcare system, care should be exercised to integrate programming across the healthcare continuum to ensure that access to culturally sensitive care is broadly enhanced.

The healthcare system needs to reflect on its capacity to meet newcomer healthcare needs and strategically respond to the healthcare needs of an increasingly diverse population. The Ministry of Health, Saskatchewan Health Authority, community clinics, and individual healthcare providers should critically examine institutionalized policies and procedures to identify how they may be contributing to the disempowerment of vulnerable groups, reflect on their capacity to serve newcomers, and respond appropriately.

## Figures and Tables

**Table 1:** Interview Questions.

Parent Interview Questions	Service Provider Interview Questions
How did you find out about accessing health care services in Canada? Please describe the first time you took your children to a health care appointment after arriving in Canada. Did your child have a health problem? What did the physician ask about? What types of tests or measurements? After that first visit to the physician, have you taken your children to see a physician again? Can you tell me about it? (Probe for location, health problem, treatment offered, follow through with treatment) Did the treatment work well—why? Was the treatment difficult to follow or work poorly? What treatments did you expect to receive for the health problem that your child did not receive? Have you sought out any other therapy or treatment? Would you like your child to receive any other therapy or treatments? Do you take your children to the public health clinic to access any services? Please describe. (Probe for immunizations, healthy child check) Has one of your children been hospitalized in Canada? Can you tell me about it? How did you manage to care for child when s/he came home? Were you offered any additional health services to help your child at home? Does your family qualify for any special health programs, such as Supplementary Health Program, Family Health Benefits or Special Support for Drug Coverage? Please describe. Which benefits do you use? (Probe for children’s dental care, eye care, prescription drug coverage, medical supplies, foot care, hearing services, chiropractic) Are some health care services easier to access than others? Please describe. What kind of changes do you think could be made to the Canadian health care system so it could provide better service to your family and other newcomers?	What barriers do you believe exist for immigrants and refugees attempting to access health care in Canada? What strategies for dealing with these barriers have you observed among immigrants/refugees? Do you believe these strategies were culturally-based? What changes could be made to reduce or eliminate these barriers? Is there anything else you can think of that could be changed to make the health care system more accessible to immigrants and refugees?

## Data Availability

The data presented in this study are available on request from the corresponding author. The data are not publicly available due to participant privacy.
